# Probing the Processes: Longitudinal Qualitative Research on Social Determinants of HIV

**DOI:** 10.1007/s10461-021-03240-w

**Published:** 2021-03-27

**Authors:** Clare Barrington, Alana Rosenberg, Deanna Kerrigan, Kim M. Blankenship

**Affiliations:** 1 Department of Health Behavior, Gillings School of Global Public Health, University of North Carolina at Chapel Hill, CB 7440, Chapel Hill, NC 27599 USA; 2 Department of Epidemiology of Microbial Diseases, Yale School of Public Health, Yale University, New Haven, CT USA; 3 Department of Prevention and Community Health, Milken Institute School of Public Health, George Washington University, Washington, DC USA; 4 Department of Sociology, American University, Washington, DC USA

**Keywords:** Social determinants, HIV, Longitudinal qualitative research, Analysis

## Abstract

Longitudinal qualitative research can provide rich understanding of the life circumstances of vulnerable groups who experience health inequities, of whether, how and why these circumstances change, and of how these circumstances and processes of change impact health. But, this rich understanding is not automatic and requires systematic and thoughtful approaches to data collection and analysis. The purpose of this paper is to describe two longitudinal qualitative studies embedded in mixed-methods studies of social determinants of HIV in the United States and the Dominican Republic. We compare these two studies to critically reflect on specific techniques that facilitate longitudinal and iterative data collection, management, and analysis, in particular the use of participant-specific matrices and analytic summaries across the distinct phases of the research. We conclude that combining cross-sectional and longitudinal analysis that engages with both themes and processes of change can contribute to improved contextualization and understanding of social determinants of HIV.

## Introduction

The World Health Organization defines social determinants of health as “the conditions in which people are born, grow, live, work and age” that are “shaped by the distribution of money, power and resources at global, national and local levels” [1]. These interrelated conditions contribute to producing health inequalities related to socio-economic status, gender, race, and ethnicity in complex and multifaceted ways. Understanding these processes requires holistic research strategies. Longitudinal qualitative research involving multiple interactions with participants over time can aid in contextualizing and improving understanding of the relationships between social determinants and health [2, 3], including HIV-related risk behaviors and care and treatment outcomes.

Longitudinal qualitative research aims to understand and contextualize processes of change, or lack thereof, over time [4, 5]. Longitudinal approaches, both planned and spontaneous, can be used across different qualitative methodologies. For example, ethnography entails extended engagement and multiple interactions over time to obtain a rich, emic perspective and produce thick description of communities [4, 6]. Narrative analysis is inherently time-oriented and can include multiple interviews to elicit stories over time in a contextualized manner [7–9]. Saldañna highlights how the orientation towards explaining processes in grounded theory methodology is a starting point for longitudinal analysis, but notes that grounded theory does not automatically include tracking processes of change through interviews over time [5].

While the use of qualitative methods in applied public health research frequently entails one-time, thematically driven interviews, there is a growing body of observational longitudinal qualitative HIV-related studies with a focus on understanding processes related to HIV vulnerability and management. With regard to HIV vulnerability, Seal et al. conducted longitudinal qualitative interviews to explore the context of risk related to HIV and other sexually transmitted infections after incarceration as part of a mixed-methods formative study and Cooper et al. used a longitudinal grounded theory approach to develop a model of the processes through which partner incarceration affects sexual risk among African-American women in the US [10, 11]. Harrison used qualitative interviews spaced 2 years apart to assess relationship dynamics among 18–24 year olds in heterosexual partnerships in South Africa [12]. Several researchers have used longitudinal narrative and thematic analysis of the process of adapting to HIV as a chronic condition, including medication adherence, clinic attendance, disclosure and stigma management [13–20]. Dang et al. assessed patient-provider dynamics through analysis of three interviews strategically timed to capture the process over time [21]. Seeley et al. analyzed longitudinal qualitative data on the relationship between HIV and household poverty in Uganda to aid in the interpretation of household survey data, highlighting the potential for longitudinal approaches to unpack the role of social determinants [22]. While these examples reflect the potential for longitudinal qualitative research to aid in understanding complex phenomenon related to HIV, there is varied and frequently limited description of the techniques used to iteratively elicit the context of experiences, manage engagement with participants, and analyze data collected over time.

Maxwell and Miller’s framework for qualitative analysis is useful for considering how to elicit and analyze longitudinal qualitative data on the social determinants of HIV [9]. Informed by the concepts of similarity and contiguity from linguistics, these authors distinguish between the process of identifying relationships based on comparison of similarities and differences, or what they refer to as “categorizing”, and the process of identifying connections within individuals and how things influence each other over time, or what they refer to as “connecting” [9]. Specifically referring to longitudinal qualitative research, Thomson and Holland’s distinction between narrative and cross-sectional analyses is consistent with Maxwell and Miller’s framework [2, 3]. While narrative analysis can trace individual processes and trajectories over time, in line with connecting, cross-sectional analysis can facilitate systematic coding of themes and concepts at a given time, in line with categorizing. Derrington’s description of “theming” also reflects the integration of categorizing and connecting to identify and track themes over time [4]. Thomson and Holland (2003) emphasize the unique contribution of both narrative and cross-sectional analyses and argue that an integrated approach facilitates an iterative process to “highlight differences and similarities within the sample, and by accumulating further rounds of analysis begin to identify the relationship between individual narratives and wider social processes” [3, p. 238].

The purpose of this paper is to describe the processes of data collection, management, and analysis of two longitudinal qualitative studies embedded in mixed-methods studies of social determinants of HIV in diverse settings. Both studies were part of the National Institutes of Health (NIH) RFA-MH-16-200: Methodologies to Enhance Understanding of HIV-Associated Social Determinants. We compare and critically reflect on the specific data collection and analysis techniques used to facilitate assessment of complex social processes and how they affect health outcomes and inequalities. We first describe the overall design of each study. We then describe our approaches to collecting and managing data over time, analytic techniques, and initial interpretation of findings.

## Methods

### Study Settings and Designs

#### JustHouHS

The Justice, Housing and Health Study (JustHouHS) is a mixed-method study to analyze the intersecting impacts of two fundamental social determinants of health in the US—mass incarceration and housing—on HIV-related sexual risks and race inequities in those risks (Table 1). The mixed-methods approach provided distinct types of data for exploring complex processes of social meaning formation and contextual influences on health outcomes and allowed us to use each type of data collection to further explore findings from the other over time. One component of the study involved the collection of survey data from a cohort of 400 low income residents of New Haven, CT, oversampled for a recent criminal justice history such that half the cohort had been released within a year of the baseline interview. Baseline and four follow-up self-administered (via Qualtrics) surveys were conducted at 6-month intervals between October 2017 and April 2020. The JustHouHS study was approved by the Institutional Review Board at Yale University. Participants received $50 for each study visit.

**Table 1:** Study settings and designs

	JustHouHS	Stigma and social cohesion
Setting	New Haven, Connecticut, United States	Santo Domingo, Dominican Republic
Purpose	Analyze the intersecting impacts of two social determinants of health—mass incarceration and housing—on HIV-related sexual risks and race inequities in those risks	Examine and contextualize the relationships between stigma, social cohesion, and HIV outcomes among female sex workers living with HIV in two distinct epidemic settings
Design and sample	Mixed-methods including longitudinal quantitative surveys (n = 400) and qualitative in-depth interviews (n = 54, selected from survey cohort) every 6 months over 2 years (up to 4 interviews). Purposive sampling based on recent incarceration history	Mixed-methods including 3 longitudinal quantitative surveys (n = 211) conducted at 12 month intervals. Three qualitative in-depth interviews (n = 20 selected from survey cohort) also at 12 month intervals. Purposive sampling based on viral suppression

Qualitative in-depth interview participants (N = 54) were purposively selected from the survey participants such that half had a recent history of incarceration while half did not. During data collection, the team reviewed gender and race composition of the qualitative cohort and used random selection from the survey cohort to achieve the same overall race and gender distribution. Seventeen participants in the qualitative sample were female and 37 were male. Thirty-four identified as African American, 9 as White, and 11 as other. Three interviewers conducted all interviews in English with a given participant; the interviewers were all middle-class, cisgender white woman in their 40s with extensive experience interviewing vulnerable populations, including those involved with the criminal justice system. Baseline interviews began in December 2017 and three rounds of follow-up interviews at 6-month intervals were conducted through January 2020. Retention was approximately 88% across waves and the number of participants per wave ranged from 46 to 54 for a total of 199 interviews. Eighty-percent of participants completed all four interviews and 94% of participants returned for at least one interview. Interviews lasted an average of about an hour, with a range of 30 min to 2.5 h. Interviews took place in the study office, located in an off-campus building close to public transportation in downtown New Haven. The JustHouHS research team included project investigators, study staff, and interviewers. The study team met two to three times a year with a Community Advisory Board, comprised of community members and those working in the fields of reentry, housing and healthcare in New Haven. The Community Advisory Board provided input into data collection and analysis.

#### Stigma, Cohesion, and HIV Outcomes Among Vulnerable Women Across Epidemic Settings

Stigma, cohesion and HIV outcomes among vulnerable women across epidemic settings (Stigma & Social Cohesion) is a mixed-methods longitudinal study integrating biologic, survey, and qualitative data (Table 1). This approach was used to obtain a holistic understanding of the social determinants of HIV outcomes among female sex workers living with HIV in Tanzania and the Dominican Republic (DR) through both inductive and deductive discovery (see Kerrigan et al. for full description of study context [23]). In this paper, we report on the Dominican site as a comparison with the JustHouHS study. The social determinants that are the focus of the study include stigma (related to HIV and sex work) and social cohesion. Participants were eligible if they were cisgender women, 18 years or older, had a confirmed HIV-positive diagnosis, and reported exchanging sex for money in the month prior to their enrollment. Using peer-referrals, 211 women were recruited for the quantitative cohort, which included 3 interviewer-administered survey interviews over 2 years at 12 month intervals. Stigma & Social Cohesion was approved by the Institutional Review Boards of the Johns Hopkins Bloomberg School of Public Health in the US and the Instituto Dermatalógico y Cirugía de Piel (IDCP) and the Consejo Nacional de Bioetica (CONABIOS) in the DR. The study protocol was also reviewed by the IDCP Community Advisory Board. All participants provided informed consent and were compensated approximately USD$ 10 per study visit.

We recruited 20 women from the survey cohort to participate in 3 in-depth interviews over 2 years, usually shortly after completing the survey. All interviews were conducted at the offices of the HIV Vaccine and Research Unit at IDCP, a research center in an accessible location with a reputation for being a safe and supportive space for sex workers. We purposively sampled based on viral load; half of the women in the qualitative sample were virally suppressed (≤ 400 copies/mL) and half were not (≥ 400 copies/mL). We used viral load as a strata in order to facilitate comparative analysis of the relationships between social determinants and viral suppression, or lack thereof. Baseline interviews were conducted in November–December 2018, second round follow-up December 2019–Jan 2020, and third round August 2020–January 2021. Retention at the second and third interviews was 100%. Baseline interviews lasted between 60 and 90 min; second and third interviews tended to run longer with some lasting over 2 h. Interviews were conducted by a Dominican cisgender woman with a background in psychology and extensive experience conducting research with sex workers. Interviews were audio-recorded and transcribed verbatim and analyzed in Spanish. The study team included the study investigators in both countries, the Dominican study coordinator and the interviewer, and research assistants in the US.

## Examples of Longitudinal Data Collection, Management, and Analysis

Interviews conducted over time can provide rich understanding of the life circumstances of vulnerable groups who experience health inequalities, of whether, how and why these circumstances change, and of how these circumstances and processes of change impact health. Below we present examples and comparative reflections on our processes of collecting, managing and analyzing, and interpreting data over time.

### Collecting Data Over Time

In reviewing our data collection processes, we identified specific considerations with regard to the instrument and interview dynamics that are unique to observational longitudinal studies embedded in mixed-methods studies with regard to: content (*what* was elicited), frequency (*when* it was elicited) and interpersonal dynamics (*how* the data was elicited and interpreted).

#### Content (What)

In both studies, baseline interview content mirrored the topic areas in the surveys with the goal of gaining more depth as well as eliciting new perspectives. In JustHouHS, these topics included housing, criminal justice involvement (self, partners, family) and experiences with and perceptions of policing, economic situation, mental and physical health, social relationships and community connections, sexual relationships, condom use, and HIV testing. In Stigma & Social Cohesion, topics included participants’ perceptions and experiences of stigma related to sex work and HIV, social cohesion, social support, and HIV care and treatment behaviors and outcomes. In both studies, baseline interviews captured current and historic information, while follow-up interviews addressed changes to these domains and probed on information shared at previous interviews, thereby allowing for a deeper understanding of topics discussed in any given interview. Across all interviews, interviewers followed the natural flow of the conversation and probed extensively for examples and experiences.

A key aspect of the follow-up interviews was the preparation beforehand. The JustHouHS research team listened to audio and read a sample of interview transcripts prior to the first follow-up to identify areas for exploration and clarification. They then discussed themes, noted places of disagreement in the interpretations of what was being discussed, highlighted places where clarification would be helpful, identified gaps and missed opportunities to gather additional details, and suggested follow-up questions. This process was most intense before the first follow-up. In subsequent waves of data collection, interviewers reviewed transcripts and the data management matrix (described below) right before the following interview to refresh their memories of what was covered and note areas to probe further.

For Stigma & Social Cohesion, given the transnational nature of the study team, data review and preparation happened at both the country and full-team levels. During baseline fieldwork, regular virtual meetings with the US and DR study teams were used to track key themes and identify areas for further probing. The full team also reviewed analytic summaries (described below) to prepare follow-up guides that built upon the previous interview as well as specific follow-up questions for each participant. For example, based on review of the baseline interviews, we revised the questions and probes about social cohesion among sex workers to improve depth of understanding of this determinant. Prior to conducting follow-up interviews, the DR-based interviewer, who conducted all interviews at all three waves, reviewed the analytic summaries and audio to prepare for each interview.

#### Frequency (When)

While both studies were fundamentally interested in obtaining a contextualized understanding of processes related to social determinants of health, neither sought to evaluate a specific intervention. In JustHouHS, there were 6 months between interviews during which there may not have been major changes to the social determinants that were being assessed, including housing and engagement with the criminal justice system. Therefore, instead of asking, “how has your living situation changed”, interviewers would start a conversation about housing, for example, by referring to a relevant detail from the earlier interview: “Last time we talked, you were unhappy with the neighborhood and said you were going to try to find a new place, how did that go?” Not only did the participant often remark that they were surprised the interviewer remembered this detail, which reinforced that they had been listened to, it also helped to create a connection between the previous and the current interview. Conversely, as participants described their current situations, they might refer to the past, or mention people or events that had been discussed in earlier interviews. This iterative process allowed for a deeper understanding of the past, as well as the present. However, interviewers also had to work to ensure that the conversations did not get diverted to the past at the expense of learning about the most recent 6 months.

While the short interval between interviews did not always allow opportunities to discuss big life changes and events in the JustHouHS study, regularly discussing participants’ experiences in their everyday lives revealed considerable insights into the ways that context constrains, enables, and gives meaning to choices and actions related to HIV. For example, at her first follow up Leah, a 25-year-old, mixed race woman, when asked whether she had an HIV test recently, responded: “Mm-hmm[yes]. Um, I think I went and got HIV tested not too long ago when I did the whole STD panel. Me and my friends, we kind of for shits and giggles just go every now and then…just, ‘Hey, you been tested lately? No? Okay, we’ll go Saturday”. In this way, she suggests that HIV testing is a part of her regular activities, something to do on “girls day out,” with no particular implication that anyone has engaged in any “undesirable” behavior. But in the next interview, she says, “Me and, um, my friend [Am], ‘cause [Am], um, she’s promiscuous and then, you know, [my] boyfriend just had his girlfriend, so we talk about going to get tested … to get, um, the HIV panel. We’re supposed … to go to Planned Parenthood. I don’t know if Planned Parenthood does HIV testing. I know they do STD testing.” And then Leah whispers, “I don’t know where we can get tested at … the last time I was tested I was in jail.” (The interviewer told her she could go to Planned Parenthood.) Perhaps Leah’s earlier response reflected a social desirability bias; as she became more familiar with the interviewer, she felt safer to admit she hadn’t been tested but wanted to know where to go. Even so, the way she discussed testing suggests that for her it is imbued with social meaning—those people engaging in “promiscuous” behaviors need testing. She challenges this idea in her first response by suggesting that she and her friends get tested on a “lark.” In the later interview, she directly connects testing to promiscuity, but still distinguishes herself from this, in what she implies is, undesirable behavior, by saying that she was last tested because she was in jail and it was part of the routine procedure there. In short, the longitudinal interviews allowed us to deepen our understanding of how changes in Leah’s perception of her risk relate to her testing behavior. They also reveal much about the social meaning of HIV testing for her; without the additional interview, we might have reached a very different conclusion about this meaning.

In Stigma & Social Cohesion, the longer (12-month) period between interviews meant that there was more opportunity for memorable changes to have occurred. However, even with this longer time-period, after the baseline interview, asking directly about changes in stigma or social cohesion did not necessarily yield rich responses. Instead, we used a grand tour question about how the last year had been for them in general and about any major changes or events in participants’ lives since the last interview [24]. Questions from the guide were anchored in the response to this grand tour question, which served as a starting point to explore experiences with stigma, social cohesion, and HIV care and treatment in a more contextualized manner. Additionally, even when there were no major changes in stigma or social cohesion, we probed on ongoing stigma management and resistance strategies to improve understanding of the experience of managing life with HIV over time. Notably, follow-up interviews generally ran longer than baseline interviews and participants usually indicated having a lot to discuss.

Most participants described some changes in their lives during the last 12 months, both positive and negative, that provided a starting point for follow-up interviews. Common examples included relationships (both beginnings and endings) and births (usually grandchildren). For example, in her second interview, Eliana indicated that the past year had been good for her with one of the major positive changes being that the partner she was starting a relationship with at the time of her first interview had moved in with her and was supporting her basic financial needs. While this was a major source of economic stress reduction for Eliana, the discussion of her relationship also led to reflection on her concerns about disclosing her HIV status to her partner, in part due to the fact that he was very jealous and had argued with her about her sex work. Eliana went on to reflect in the second interview about the pros and cons of staying in this relationship, which facilitated a contextualized understanding of how anticipated HIV stigma and enacted sex work stigma, among other factors, influenced her decisions. By the third interview, they had broken up and stopped all communication. Eliana expressed that she hoped to find a partner who also lived with HIV and with whom she could live openly about her condition as she believed this would be mutually beneficial for their wellbeing, including supporting each other with treatment adherence and nutrition. She also indicated wanting to leave sex work as she felt it was not good for her physical or mental health at this point in her life, but she continued due to her economic necessity. Having the three interviews allowed us to follow the progression of Eliana’s relationship and the ways it affected her health and wellbeing.

#### Interpersonal Dynamics and Evolving Narratives in the Research Settings (How)

Both studies took measures to lessen the possibility that participants felt judged, surveilled, or interrogated as they were asked about personal and stigmatized topics over time. In both studies, the same interviewers interviewed the same participants at each wave, which provided the opportunity to develop rapport, comfort, and trust. Both study teams also maintained an open-door policy at the research sites that allowed participants to check-in and informally share updates between interviews with interviewers and other study staff. This facilitated retention as well as an ongoing relationship between participants and interviewers. It also allowed participants to talk when they had something to say, not just in the interview setting. When relevant to the study, these interactions were documented by interviewers and included in participant’s study files. Across studies, participants expressed that they enjoyed the interviews and looked forward to coming back. They mentioned that other people didn’t usually ask them these kinds of questions and that they told the interviewer things they rarely or never talked about. These dynamics of rapport and sharing observed in both studies are examples of the expected strengths of interviewing people repeatedly over time that facilitated more depth and contextualized understanding of social determinants.

However, the development of the relationship also meant that the interviewers’ and participants’ impressions of each other, and the accompanying dynamics of power that each held, influenced the construction of the narrative, as has been noted by others [3–5, 7]. The highly stigmatized topics of study, namely criminal justice involvement, HIV, and sex work, contributed to this possibility. In Stigma & Social Cohesion, Yenny was incarcerated between her first and second interview. Upon her release, she immediately contacted the research team and while she was eager to conduct her follow-up interview, she also expressed shame and reluctance to talk about what had led to her incarceration, fearing it had damaged her reputation with the team. In both studies, it was important to maintain detailed documentation of these relationships over time to avoid relying on the partial depiction of these relationships in the transcripts.

In both studies, actions were taken to maximize participants’ control of their narratives. In Stigma & Social Cohesion, baseline interview questions were tailored to probe on women’s perceptions and understandings of their viral suppression status, which was known as it was used to determine the sample. However, the most recent viral load was not available to the interviewer prior to follow-up interviews, leaving participants to share their perceived understanding of their viral load and also what they felt comfortable sharing during the interview, regardless of whether it reflected their “biological reality”. Likewise, in JustHouHS, although the team had survey data about participant’s sexual relationships and practices, which at times may have differed from what they discussed in interviews, interviewers did not reference or try to “verify” survey findings during interviews. Key survey variables were included on the matrix (described below) and served two purposes: one, they were reviewed as part of general overview of participant circumstances during data analysis, and two, they were compiled in the matrix for easy access for planned mixed methods analyses.

While both study teams aimed to establish rapport and allow participants to control their own narratives, the study settings had some differences that also affected how the research was done. The research sites in Stigma & Social Cohesion had previously participated in intervention research with sex workers. Beyond their rapport with the interviewers, through participation in these past studies, some participants had trusted relationships with the larger study staff and considered the study sites to be safe places where they could expect to be treated respectfully. Many participants were still active in community mobilization efforts with other sex workers living with HIV established in the previous intervention research projects in both countries and therefore also had connections to other women in the cohort.

Several participants in the JustHouHS study made references to New Haven being a “research town” and conveyed distrust of studies done by the university in the realm of HIV. In addition to the open-door policy at the study site, study staff addressed this potential lack of trust by becoming well-versed in community resources and making referrals when possible. The team also published an annual community report to share findings with participants, which allowed for transparency of data and underscored researchers’ intention to use the data to expose systemic problems faced by participants.

#### Managing and Analyzing Data Over Time

Both studies used a combination of cross-sectional and longitudinal approaches to analyze the large amounts of data collected over time, including matrices, analytic summaries, indices, coding, and memos (Table 1).

#### Case-Based Matrices and Analytic Summaries

Both studies created specific analytic tools to facilitate data management over time that were essential to facilitating the iterative data collection and analysis processes central to longitudinal approaches. The JustHouHS team created a *case-based matrix* (Table 2) with comprehensive summaries of participant data at each wave. In addition to facilitating ongoing data collection and quick familiarization with data at each time point, these matrices served as a tool for connecting data across time and identifying areas for follow-up. The team also created a master topical matrix combining information from the survey and the case-based matrices and organized with separate tabs for each substantive domain, allowing the identification of themes, and comparison of context and experiences across participants, over time.

**Table 2:** Techniques for longitudinal and cross-sectional analysis of in-depth interview data

Techniques	JustHouHS	Stigma and social cohesion
Case-based tracking and synthesis documents Facilitated data management Enabled quick familiarization with data at each time point Aided systematic preparation of follow-up questions	A *matrix* was created for each participant summarizing data at each of the four waves including demographic information, survey data, and interview summaries Organized by the primary study domains (e.g. housing, economic situation, health, criminal justice involvement, substance use, partners and sex, HIV) Contributed to a master topical matrix that combined information from different cases, with separate tabs for each substantive domain, allowing the identification of themes, and comparison of context and experiences across participants, over time	An *analytic summary* was created for each participant that included socio-demographic data and viral suppression status, and the following sections with space for each of the three interviews Contextualized summary of HIV narrative including diagnosis, family and relationship dynamics, and managing life with HIV Key quotes and themes (inductive and deductive) used to develop codebook Summary of content of topical modules included in the interview guide at each wave (e.g. mobility, perceptions of long acting injectable ART, mindfulness) Follow-up questions to clarify content from previous interviews and probe on emergent areas
Indexing Identified large sections of text by topic Facilitated easy retrieval of information	20 indices related to main study domains applied to transcripts in Nvivo Facilitated development of codes and writing of analytical memos	Section within analytic summaries indexed data from topic-specific modules (long-acting injectable anti-retroviral therapy (ART), mindfulness, mobility). This facilitated efficient review of topical information while keeping the data within the overall context of the participant Example: mixed methods analysis of perceptions and attitudes of long-acting injectable ART at baseline [26]
Coding Labeled thematic content systematically using software	Used in conjunction with indices Example: for an analysis of the residential experiences of people returning from prison, excerpts labeled with the index “housing” were further coded with sub-codes related to types of residences (halfway houses, homeless shelter, etc.) in Nvivo	Codes were developed to label sections of the HIV narrative elicited in baseline interviews including diagnosis, disclosure, initiating care/treatment. Coding these chapters using Atlas.ti facilitated comparative analysis of HIV trajectories based on viral suppression status across participants We plan to use time-focused codes to systematically track processes related to stigma and social cohesion
Memo writing Facilitated analysis and interpretation of original research questions and emergent themes Bridged analytic and interpretive processes	Could be specific to one transcript, participant, group of participants, or thematic Facilitated sharing analytic thinking prior to research team meetings and summarized ideas generated during meetings Assisted with more deeply understanding the data Example: in an analysis of gender and HIV risk, material indexed at the intersection of relationships, HIV, and sex was used to write participant-specific analytical memos related to themes of interest	Within the analytic summary, a memo-within-a-memo to describe emerging ideas regarding processes related to stigma and social cohesion over time *within* participants Thematic memos will be used to facilitate cross-sectional and longitudinal analysis of key themes *across* participants

In Stigma & Social Cohesion, following baseline interviews, the team developed an *analytic summary template* (Table 2) to facilitate both connecting and categorizing approaches in early analysis and to systematically inform subsequent data collection. The analytic summary is a living document that includes basic socio-demographic data and baseline viral load followed by these sections: (1) an HIV narrative summary; (2) key quotes and themes related to stigma and social cohesion; (3) summary of topical modules included at specific waves of data collection (including mobility, mindfulness, and perceptions of long acting injectable anti-retroviral treatment); and follow-up questions for future interviews. By including both contextualized narratives as well as topical content, this document facilitated holistic tracking of stories and themes over time. Each section includes a separate space for each of the 3 waves of data collection to facilitate both cross-sectional and longitudinal analysis.

#### Indexing and Coding

In both studies, *indexing and coding* were also used for both cross-sectional and longitudinal analyses. Indexing, or chunking out large sections of text related to domains of interest, facilitated easy retrieval of information within and across time points. For JustHouHS, the research team identified 20 indices related to the main domains of interest. These indices allowed for the easy retrieval of excerpts relevant to a specific domain and were thus useful in two ways. First, they facilitated the application of sub-codes/analytical codes. Second, they facilitated writing of analytical memos. For example, in an analysis of gender and HIV risk, material indexed at the intersection of relationships, HIV, and sex was used to write participant-specific analytical memos related to themes of interest [25]. As noted above, indexing was included as a section in the analytic summaries in the Stigma & Social Cohesion for cross-sectional analysis of specific topics to summarize both inductive and deductive themes at each time point. Including indexing in the analytic summary facilitated efficient review of topical information while keeping the data within the overall HIV narrative and context of the participant. Coding was used to facilitate analysis of retrospective HIV narratives using baseline data and will be used to track themes over time to facilitate comparison across participants, for example extracting text on examples of stigma and social cohesion over time.

#### Analytic Memos

The techniques described above contributed to data management, restructuring, and early analysis. As our teams shift into more extensive analysis and interpretation, *writing through memos* has been critical to facilitating both cross-sectional and longitudinal analyses that respond to the original research questions as well as emerging themes and directions of inquiry. For JustHouHS, these memos could be specific to one transcript, multiple transcripts of one participant, or grouped participants, or thematic. Memos facilitated sharing analytic thinking prior to research team meetings and summary of ideas generated during meetings. Memo writing assisted with more deeply understanding the data than coding, matrices, or reading alone and is a critical component of the longitudinal analysis process. Following the second interview in Stigma & Social Cohesion, the team added a section to the end of the analytic summary template summarizing interpretation of processes of stigma and social cohesion. This section serves as a memo-within-a-memo to facilitate analysis of social determinants over time.

### Interpreting Qualitative Data Collected Over Time

In these two studies, we used longitudinal qualitative designs to develop contextualized understandings of how social determinants shape health outcomes. In this final section, we reflect on the ongoing interpretive process to address the aims of each study.

### Processes and Understanding Pathways of Stigma and Social Cohesion

Based on the team’s prior research [27], the hypothesis of the quantitative aim of Stigma & Social Cohesion was that social cohesion would mediate the influence of stigma on HIV outcomes. The qualitative aim was to contextualize the relationships and probe on the processes between stigma, social cohesion, and HIV outcomes. The longitudinal design has allowed for obtaining a deeper and contextualized understanding of dynamic processes related to experiencing and managing stigma, including insights into the role of social cohesion, which would not be possible in a qualitative cross-sectional study or a stand-alone, hypothesis-driven quantitative assessment.

Echoing past work, we have found that while stigma is a constant and steady presence in women’s lives, responses and resistance to stigma are dynamic. Most women describe individual-level processes of normalization—mainly, trying to not think about stigma—as a way to minimize its effects on their wellbeing; these processes were often connected to limiting social engagement and not disclosing involvement in sex work or HIV status. Some women found support in groups of sex workers living with HIV; paradoxically, disclosure in these spaces is required to facilitate the connection that is foundational to processes of constructing social cohesion [28].

One participant in the DR, Reina, highlights how these processes of stigma and social cohesion are connected to her wellbeing over time. Reina retrospectively described in her baseline interview that she initially denied her HIV status and didn’t engage in care but after a few years accepted it, started anti-retroviral therapy, and became suppressed; she was virally suppressed at her baseline and follow-up interviews (self-report). At baseline, she indicated that her mother, the only person in her family who knew her HIV status, distanced herself when she found out, which caused Reina to feel depressed and periodically stop her medication. In her first follow-up interview, when asked about the most important thing that had happened in the last year, she mentioned her improved relationship with her mother, which represented what Saldaña refers to as epiphanies, or “moments that alter future thought and action” [29, p. 51]. They had established daily communication and Reina emphasized how important it was to know that she had someone who was concerned about her. Reina still did not speak explicitly about her HIV with her mother, however, reflecting how she continued to manage stigma even in the context of their improved relationship. In the third interview, the interviewer probed on what Reina thought had caused the change in her mother, which is an example of how longitudinal interviews can be used to obtain more depth of understanding about past events, not just change over time. Reina indicated that this was something she continually reflected upon but she did not have a definitive response and had just accepted this as a positive change. While they no longer sustained daily communication at the time of the third interview, which Reina attributed to her limited access to the internet, she still felt supported by her mother. The father of Reina’s children had recently passed away following a health crisis due to diabetes, during which time Reina cared for him since he had no other family. She relied heavily on her mother and sisters to support her during this time, including giving advice on how to care for him and accompanying her as she took him to medical appointments.

While Reina said in all of her interviews that she kept to herself to avoid being stigmatized, she was actually one of the most socially engaged participants in the study, but always in a very selective way to avoid stigma or involuntary disclosure to her children. At the community level, at baseline, Reina shared that she had been denied participation in the parent’s association of her youngest child’s school as well as the board of her neighborhood association, where she was a lifelong resident. At her 2nd follow-up, she had changed her child to a new school and joined the parent association, but she had not pursued involvement in the neighborhood group out of fear that it could lead to her children finding out that she was living with HIV. In the third interview, she commented that changing schools had been hard on her son, but firmly insisted that she had no option in order to protect him from “hearing things he should never have to hear”. Reina’s reflection on her act of resistance in the third interview provided the opportunity to deepen our understanding of how she dynamically resisted or managed stigma based on her assessment of risk and priority, a process that could only be followed through multiple interviews.

#### Meaning and Context Around Social Determinants of HIV Risk

For JustHouHS, the effort to build an understanding of context and its relation to HIV related sexual risk has been an iterative process. As such, the team has reflected on whether the interviews might as easily be considered “repeated” as they are “longitudinal.” Each interview became, simultaneously, an opportunity to enrich our understanding of how participants assign and convey meaning to both the quotidian details of and the major events in their lives, and how these meanings might reflect their experiences of a context shaped by mass incarceration and housing. As this understanding expanded over time, the team developed explanations for how housing and mass incarceration intersect to produce HIV-related sexual risks.

Participants’ answers to specific questions about their HIV risk were often quite short. Even so, they revealed how embedded in the meanings they gave to “protection” and “risk” were judgments about who was at risk and, typically, why *they* were not at risk. Desiree, for example, was pregnant with her fourth child at the time of the baseline interview. She stated that she always used condoms to “stay safe.” But her pregnancy (which she did not attribute to a failed condom), and other contextual information she shared about her life and her partner over the course of the study, demonstrated how she made a determination of whether, with whom, and under what conditions unprotected sex is “safe” for her.

While repeated interviews helped to better understand the meanings respondents gave to HIV-related risk and protective behaviors, it also became clear that the team didn’t need to have extensive and explicit discussions with respondents about their protective strategies or their views on their risks. Over time their descriptions of their housing and related living arrangements and their struggles to maintain or improve them, of their own evolving histories with the criminal justice system and those of their loved ones, of life in neighborhoods under the pervasive gaze of the extended carceral state, provided a clear picture of their HIV-related risks. Desiree, for example, had a Section 8 housing voucher that provided her a government subsidy that made her rent affordable, gave her some degree of housing stability, and ensured her highest priority: caring for her four children. Her partner, also the father of her two youngest children, with whom she hoped to have a long-term relationship, officially lived with his mother, in part because, in light of his criminal justice history, this arrangement protected her access to stable housing. But her status as an independent leaseholder had implications for his residential movements between his mother’s and her place throughout the study period, sometimes meaning that he would not be in contact with Desiree for weeks at a time. This, in turn, influenced her potential HIV risk. In this way, and as we elaborate further in the article in this issue [25], for women like Desiree, context and not their behaviors shaped their risk for HIV.

## Conclusions

Longitudinal qualitative research has the potential to improve our understanding of complex social processes and relationships that can contribute to health-related behaviors and outcomes. By using longitudinal approaches in the JustHouHS and Stigma & Social Cohesion studies, we are expanding our contextualized understanding of social determinants of HIV. There is no correct or best way to analyze any qualitative data, cross-sectional or longitudinal, and the research questions guiding the study will always be the starting point for selecting an analytic approach [5, 29]. In addition to using standard qualitative data collection and analytic tools, both studies described in this paper relied heavily on tools for managing data and facilitating iterative analysis during data collection, the case-based matrix in JustHouHS and the analytic summary in Stigma & Social Cohesion. These tools aided in managing the data, summarizing initial findings, and facilitating follow-up. It is important to highlight the contribution of these living, participant-specific documents in the methods sections of papers to reflect how the longitudinal process is used to advance understanding of complex phenomenon beyond what can be done with a single interview.

The opportunity to “attend to temporality” is both a strength and a challenge of longitudinal analysis [30, 31]. Ryan et al. raise the concern of whether follow-up interviews could undermine the interpretation of earlier interviews as “repeat interviews highlight the contingency of individual accounts and interpretations” [30]. Coming from a constructivist perspective, rather than seeing the potential for undermining, we consider repeat interviews as a way to broaden, expand, and fill in gaps, while also, at times, complicating the interpretation, as reflected in our examples. Connected to time is the notion of change, which both study teams grappled to assess, especially since we were not evaluating specific programs or policies. We found that linking back to past interviews and asking indirect and broader questions, rather than only direct questions about change, helped to elicit reflection on change and time within participants narratives. We also found that learning about quotidian practices and relationships aided in our contextualized understanding of the social determinants we aimed to study regardless of whether they had changed.

Along with the opportunities for depth and context, the challenges associated with qualitative research with regard to reflexivity, power, and control of the narrative are all intensified in the context of longitudinal studies. In both studies, we aimed to situate our work within ongoing community-driven processes and resources and to create a safe space for engagement, reflection, and collaborative learning [32]. While participants indicated appreciating the process, it is important to be thoughtfuld and reflexive about these dynamics in the interpretation of findings. Miller reflects on the potential challenge of participants “losing the plot”, referring to the experience of participants not feeling that their experiences align with normative “public” narratives and discourses [7]. This is an important consideration for studies of social determinants of stigmatized topics, such as HIV, incarceration and sex work, where participants may already be struggling to line their experiences up with “public” narratives and make sense of their experiences in a linear and coherent manner [7]. Further reflections on the opportunities and challenges related to the dynamics between interviewers and participants and a larger reflection on reflexivity warrants a paper on its own to advance the field of longitudinal qualitative research.

In summary, we believe there is great potential for increasing and improving the use of longitudinal qualitative approaches in public health scholarship to expand understanding of the dynamic process that shape health inequities and provide more nuanced and actionable inputs for program and policy design.
