# Correction to: Probing the Processes: Longitudinal Qualitative Research on Social Determinants of HIV

**DOI:** 10.1007/s10461-021-03444-0

**Published:** 2021-09-13

**Authors:** Clare Barrington, Alana Rosenberg, Deanna Kerrigan, Kim M. Blankenship

**Affiliations:** 1 Department of Health Behavior, Gillings School of Global Public Health, University of North Carolina at Chapel Hill, CB 7440, Chapel Hill, NC 27599 USA; 2 Department of Epidemiology of Microbial Diseases, Yale School of Public Health, Yale University, New Haven, CT USA; 3 Department of Prevention and Community Health, Milken Institute School of Public Health, George Washington University, Washington, DC USA; 4 Department of Sociology, American University, Washington, DC USA

## Correction to: AIDS and Behavior 10.1007/s10461-021-03240-w

The article “Probing the Processes: Longitudinal Qualitative Research on Social Determinants of HIV”, written by Clare Barrington · Alana Rosenberg · Deanna Kerrigan · Kim M. Blankenship was originally published electronically on the publisher’s internet portal on 27th March 2021 without open access. With the author(s)’ decision to opt for Open Choice the copyright of the article changed on 6th August 2021 to © The Author(s) 2021 and the article is forthwith distributed under a Creative Commons Attribution.

The original article has been corrected.

